# Long-term use of clopidogrel versus ticagrelor or prasugrel in patients with acute myocardial infarction after percutaneous coronary intervention

**DOI:** 10.1371/journal.pone.0278993

**Published:** 2023-02-23

**Authors:** Yuri Kim, Myung Ho Jeong, Minjeong An, Kyunghoon Cho, Youngjoon Hong, Juhan Kim, Youngkeun Ahn

**Affiliations:** 1 College of Nursing, Chonnam National University, Gwangju, Korea; 2 Department of Cardiology, Chonnam National University Hospital and Medical School, Gwangju, Korea; Baylor Scott and White, Texas A&M College of Medicine, UNITED STATES

## Abstract

**Background and objectives:**

To compare the long-term clinical outcomes of dual antiplatelet therapy (DAPT) with clopidogrel and DAPT with ticagrelor or prasugrel in patients with acute myocardial infarction (AMI) who underwent coronary intervention.

**Methods:**

Between November 2011 and December 2015, a total of 13,104 patients with AMI were enrolled in the Korea Acute Myocardial Infarction Registry-National Institutes of Health (KAMIR-NIH) registry. Among them, 4,696 patients who received DAPT for more than 24 months were categorized into two groups: the clopidogrel group (n = 4,053) and ticagrelor or prasugrel group (n = 643). Propensity score matching (PSM) was used to reduce the bias due to confounding variables. Following PSM, the impacts of P2Y_12_ inhibitors on the clinical outcomes in both groups were compared during a 36-month clinical follow-up period.

**Results:**

There were no significant differences in clinical outcomes in terms of cardiac death (7.1% vs. 9.7%, p = 0.101), stroke (1.4% vs. 1.0%, p = 0.436), major bleeding (0.5% vs. 0.8%, p = 0.478), major adverse cardiac events (MACE) (21.6% vs. 20.5%, p = 0.626), and net adverse cardiac event (NACE) (22.1% vs. 21.3%, p = 0.731) between the groups. The ticagrelor or prasugrel group had a lower incidence of recurrent percutaneous coronary intervention (PCI) (12.2% vs. 7.6%, p = 0.006) than the clopidogrel group. However, no differences were observed in the cumulative incidences of 3-year NACE between the ticagrelor or prasugrel and clopidogrel groups.

**Conclusions:**

Cumulative incidences of long-term NACE did not differ between the two groups. Therefore, the type and duration of DAPT should be customized for each patient with AMI.

## Introduction

Dual antiplatelet therapy (DAPT), consisting of a combination of aspirin and P2Y₁₂ inhibitor, is the cornerstone of pharmacological treatment aimed at preventing atherosclerotic complications in patients with coronary artery disease [1]. Because the inhibition of platelet aggregation using antiplatelet agents is crucial for the treatment of patients with acute myocardial infarction (AMI), maintaining antiplatelet therapy for 12 months after the intervention is recommended in patients with AMI [2]. However, 85% of prodrug clopidogrel is inactive. As the rest of clopidogrel is activated via oxidation by liver cytochrome P-450, the onset of drug efficacy is slow, and there are large individual differences in platelet reactions, resulting in little or no platelet inhibitory effect in some patients [3]. In particular, CYP2C19 gene polymorphism, which occurs in more than 30% of white individuals, 40% of black people, and 55% of the East Asian population, reduces pharmacokinetic responses to clopidogrel from about 25% to 33% [4]. These factors impact the clinical outcomes of clopidogrel, including stent thrombosis, in patients with AMI, highlighting the need for new drug development [3]. New antiplatelet agents such as ticagrelor and prasugrel have more consistent and potent platelet inhibitory effects compared with clopidogrel [5].

Both the American College of Cardiology/American Heart Association (ACC/AHA) and European Society of Cardiology (ESC) guidelines recommend that aspirin therapy be used in combination with ticagrelor or prasugrel, rather than clopidogrel, in patients with Non-ST segment elevation myocardial infarction (NSTEMI) or ST-segment elevation myocardial infarction (STEMI) with no contraindications [6, 7]. However, compared with clopidogrel, ticagrelor and prasugrel reduce the risk of ischemic events and increase bleeding risk [8, 9]. In addition, the ACC/AHA and ESC guidelines recommend maintaining DAPT for at least 12 months in patients with AMI [6, 7].

A domestic SMART-DATE study on DAPT’s maintenance period compared patients who took DAPT for more than 6 and 12 months after percutaneous coronary intervention (PCI) [10]. Myocardial infarction occurred more frequently in the 6-month group than in the 12-month group [10]. The Korean Society of Myocardial Infarction also recommends DAPT for at least one year and prolonged DAPT in high-risk patients [11]. When comparing DAPT at 3 months and 12 months in the SMART-CHOICE and TICO trial studies, there was no difference in prognosis between the two groups [12, 13].

In the PEGASUS-TIMI 54 trial, DAPT with aspirin and ticagrelor was used to treat patients with AMI who were then observed for up to 36 months [14]. The relative risk of death from cardiovascular causes, myocardial infarction, and stroke in patients treated with aspirin and ticagrelor was significantly lower compared with the placebo group [14]. The THEMIS trial suggested prolonged DAPT in diabetic patients [15]. Therefore, the duration of DAPT in patients with myocardial infarction after PCI must be determined.

Since previous studies on DAPT were mainly short-term studies, this work aims to investigate the clinical outcomes of DAPT in patients with myocardial infarction who maintained DAPT for more than 24 months after hospitalization without halting or changing the medication.

## Methods

### Study design and population

In the present study, data were obtained from the Korea Acute Myocardial Infarction Registry-National Institute of Health (KAMIR-NIH) registry. The KAMIR-NIH study is a prospective, multicenter, observational, and web-based cohort study aiming to determine the prognosis and indicators for treatment strategies in patients with AMI who were registered at 20 major PCI centers in Korea from November 2011 to December 2015. This trial was supported by the Korea NIH [16]. This study was approved by the Institutional Review Board of Chonnam National University Hospital (CNUH-2011-172).

From November 2011 to December 2015, a total of 13,104 patients with AMI who underwent PCI with a drug-eluting stent (DES) were enrolled in the KAMIR-NIH, a Korean acute myocardial infarction registration study. Among them, 4,696 patients who maintained DAPT without stopping or changing the drug for more than 24 months were divided into two groups. We also incorporated new antiplatelet agents such as ticagrelor and prasugrel to compare with clopidogrel, resulting in a clopidogrel group (n = 4,053) and ticagrelor or prasugrel group (n = 643).

### Definition and clinical endpoint

**Fig 1:**
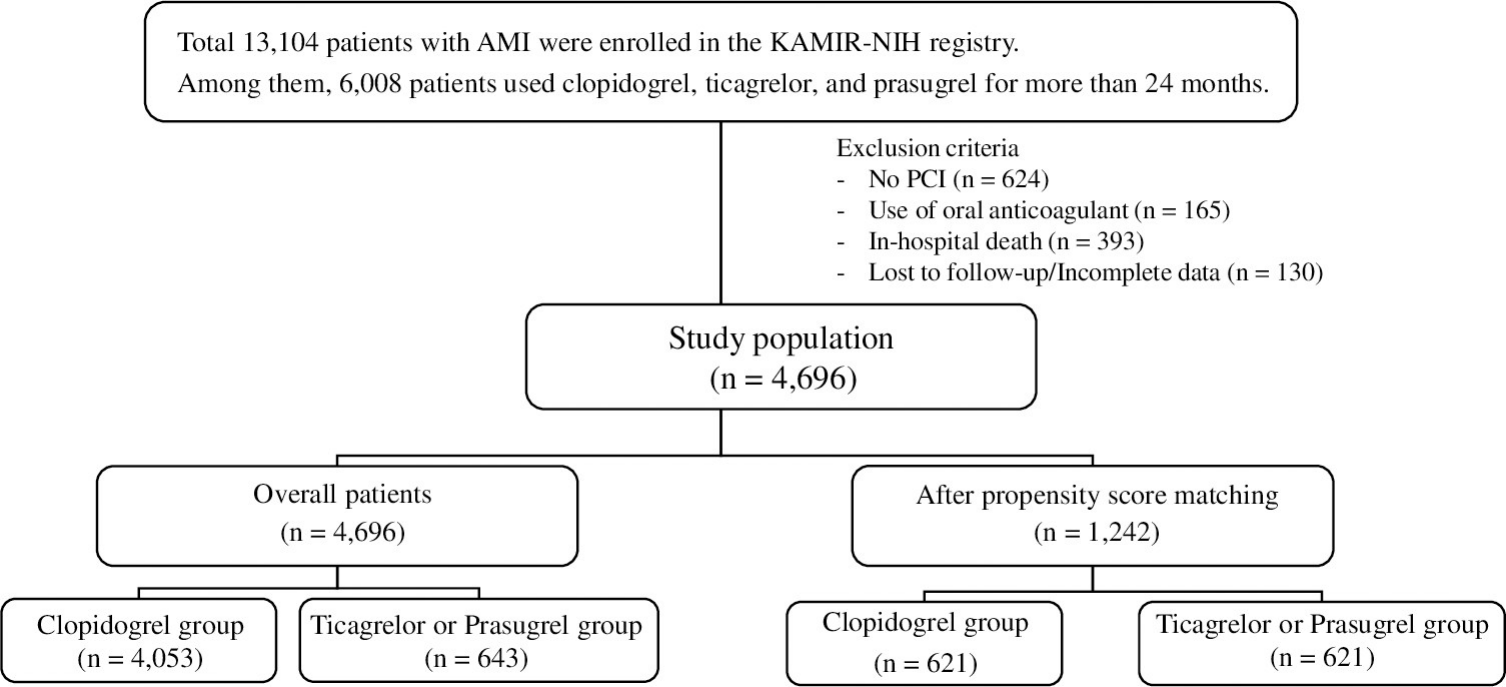
Study flow chart. AMI: acute myocardial infraction, KAMIR-NIH: Korean Acute Myocardial Infarction Registry National Institutes of Health, PCI: percutaneous coronary intervention.

Antiplatelet agents, such as clopidogrel at a loading dose (LD) of 300 or 600 mg, ticagrelor at an LD of 180 mg, or prasugrel at an LD of 60 mg, were administered in combination with aspirin at an LD of 300 mg in all patients before PCI. After PCI, all patients received clopidogrel at 75 mg once daily, prasugrel at 10 mg once daily, or ticagrelor at 90 mg twice daily in combination with aspirin at 100 mg once daily. PSM was used next to reduce the bias due to confounding variables (Fig 1).

Information on general characteristics, including sex, age, body mass index (BMI), Killip class, comorbidities [e.g., hypertension (HTN), diabetes mellitus (DM), dyslipidemia (DL), previous myocardial infarction, angina, heart failure (HF), and cerebrovascular accident (CVA)], and smoking rate were collected. Clinical characteristics were recorded following hematological tests and echocardiographic examinations to measure left ventricular injection fraction (LVEF) conducted during hospitalization. I lesion characteristics in coronary artery angiographic findings were classified using the ACC/AHA classification [17]. The rate of perfusion through coronary artery lesion was classified according to the Thrombolysis in Myocardial Infarction (TIMI) flow grade [18].

The primary efficacy endpoint this study was defined as the cumulative incidence of major adverse cardiac events (MACE), including cardiac death, nonfatal MI, repeat PCI, coronary artery bypass graft (CABG), and ischemic stroke, during 3 years of clinical follow-up. The primary safety endpoint was defined as the incidence of TIMI major bleeding during 3 years of clinical follow-up.

The secondary endpoints were defined as the incidences of net adverse cardiovascular events (NACE), including TIMI major bleeding, cardiac death, nonfatal MI, repeat PCI, CABG, and ischemic stroke, during 3 years of follow-up. Bleeding events were classified as major or minor according to the TIMI scales [19].

### Statistical analysis

Continuous variables are presented as mean ± standard deviation and were analyzed using the student’s t-test. Categorical variables are expressed as numbers and percentages, and they were analyzed using the Chi-square test or Fisher’s exact test. To minimize the effect of selection bias between the clopidogrel and ticagrelor or prasugrel groups, propensity scores (PSs) were estimated using a multivariate logistic regression model for baseline clinical, angiographic, and procedural characteristics and prescribed medications. Multivariate logistic regression was performed with the independent variables for all individual outcome components. Only variables with a p-value < 0.1 in the univariate analysis were included in the multivariate model. The C-statistic of the PS model was 0.788. Next, using the nearest neighbor matching method, each patient in the clopidogrel group was matched with one patient in the ticagrelor or prasugrel group according to the PS. Patients were matched using a caliper width of 0.01 of the standard deviation of the PS logit. Baseline clinical, angiographic, and procedural characteristics and prescribed medications were compared between the two propensity-matched groups. To assess the predictors of mortality and major cardiac events, the hazard ratio (HR) and 95% confidence interval (CI) were calculated using univariate Cox regression analysis and multivariate Cox regression analysis for variables with p-values < 0.1. The results are presented as adjusted hazard ratios with 95% confidence intervals. Kaplan–Meier analysis was performed to compare MACE, and the log-rank test was used to test the differences between the survival curves. All analyses were two-tailed, and statistical significance was set at p ≤ 0.05. All analyses were performed using SPSS software (version 25.0; IBM Co., Armonk, NY, USA).

## Results

### Baseline characteristics and medication

The proportion of male subjects, mean age, and the Killip class measured at admission were higher in the clopidogrel group than in the ticagrelor or prasugrel group, and the incidence of dyspnea was more frequent in the clopidogrel group than that in the ticagrelor or prasugrel group. There were more patients with a history of HTN and CVA and fewer with a history of MI and smoking in the clopidogrel group than in the ticagrelor or prasugrel group. After PSM, the incidence of dyspnea was higher in the clopidogrel group than in the ticagrelor or prasugrel group, and there were no differences in sex, age, and comorbidities between the two groups (Table 1).

**Table 1:** Clinical characteristics of the clopidogrel and ticagrelor or prasugrel groups.

	Overall patients			After propensity score matching		
	(n = 4,696)			(n = 1,242)		
Variables	Clopidogrel group (n = 4,053)	Ticagrelor or prasugrel group (n = 653)	p-value	Clopidogrel group (n = 621)	Ticagrelor or prasugrel group (n = 621)	p-value
**Baseline clinical characteristics (%)**						
Male	2,909 (71.8)	509 (79.2)	<0.001	480 (77.3)	491(79.1)	0.450
Age (years)	65.56 ± 12.70	61.45 ± 12.77	<0.001	61.15 ± 12.97	61.45 ± 12.67	0.682
Systolic blood pressure (mmHg)	131.83 ± 28.93	127.68 ± 27.83	0.001	132.84 ± 27.72	129.47 ± 27.74	0.054
Diastolic blood pressure (mmHg)	79.16 ± 17.44	78.33 ± 16.67	0.259	80.97 ±17.94	78.23 ± 16.59	0.097
Heart rate (beats/min)	78.65 ± 18.76	78.85 ± 18.47	0.798	78.32 ± 17.88	78.79 ± 18.44	0.651
BMI (kg/m^2^)	23.82 ± 3.411	24.41 ± 3.37	<0.001	24.40 ± 3.29	24.40 ± 3.78	0.988
Typical chest pain	3,469 (85.6)	559 (86.9)	0.364	537 (86.5)	541 (87.1)	0.737
Dyspnea	1,021 (25.2)	109 (17.0)	<0.001	135 (21.7)	104 (16.7)	0.026
Killip class > 2	527 (13.0)	66 (10.3)	0.052	63 (10.1)	64 (10.3)	0.925
STEMI	1,944 (48.0)	321 (49.9)	0.356	302 (48.6)	314 (50.6)	0.496
Hypertension	2,218 (54.7)	332 (50.1)	0.028	330 (53.1)	313 (50.4)	0.334
Diabetes mellitus	1,479 (36.5)	236 (36.7)	0.918	242 (39.0)	230 (37.0)	0.483
Dyslipidemia	481 (11.9)	62 (9.6)	0.101	60 (9.7)	59 (9.5)	0.990
Previous MI	346 (8.5)	76 (11.8)	0.007	69 (11.1)	70 (11.3)	0.928
Previous angina	424 (10.5)	59 (9.2)	0.319	54 (8.7)	57 (9.2)	0.765
Previous HF	62 (1.5)	10 (1.6)	0.961	16 (2.6)	10 (1.6)	0.234
Previous CVA	306 (7.5)	33 (5.1)	0.028	34 (5.5)	32 (5.2)	0.800
Smoking	2,257 (55.7)	408 (63.5)	<0.001	374 (60.2)	392 (63.1)	0.293
**Concomitant medication (%)**						
CCB	270 (6.7)	42 (6.5)	0.902	41 (6.6)	39 (6.3)	0.921
Beta blocker	3,436 (84.8)	545 (84.8)	0.991	532 (85.7)	526 (84.7)	0.632
ACEI	1,955 (48.2)	229 (35.6)	<0.001	226 (36.4)	216 (34.8)	0.553
ARB	1,360 (33.6)	315 (49.0)	<0.001	300 (48.3)	309 (49.8)	0.609
Statin	3,789 (93.5)	613 (95.3)	0.072	593 (95.5)	519 (95.2)	0.788
**Echocardiography findings**						
LVEF (%)	51.41 ± 11.11	51.82 ± 10.89	0.379	51.59 ± 11.19	51.84 ± 10.89	0.690
**Laboratory findings at admission**						
Hemoglobin (g/dL)	13.55 ± 2.16	13.96 ± 2.11	<0.001	13.92 ± 2.19	13.95 ± 2.11	0.810
Platelet count (10^3^/μL)	230.60 ± 68.04	230.67 ± 60.70	0.981	229.48 ± 64.17	230.95 ± 60.46	0.678
Creatinine (mg/dL)	1.19 ± 1.29	1.11 ± 0.97	0.159	1.23 ± 1.75	1.12 ± 0.98	0.144
CK-MB (ng/mL)	107.82 ± 158.48	104.19 ± 112.63	0.578	116.37 ± 189.99	104.88 ± 113.15	0.198
Troponin I (ng/mL)	49.58 ± 100.34	50.05 ± 73.83	0.915	52.87 ± 101.11	49.97 ± 72.18	0.582
Hs-CRP (mg/dL)	1.57 ± 6.36	1.24 ± 3.25	0.405	1.15 ± 2.96	1.24 ± 3.28	0.694
NT-pro-BNP (pg/mL)	3128.79 ± 10096.22	2962.40 ± 6816.70	0.898	2439.42 ± 6526.94	2972.98 ± 6928.64	0.268
ARU (units)	467.35 ± 72.72	452.71 ± 74.57	0.067	467.51 ± 72.35	452.52 ± 74.80	0.466
PRU (units)	224.60 ± 97.26	159.96 ± 115.51	0.016	210.16 ± 98.63	189.38 ± 114.98	0.103

Data are presented as number (%). Data are presented as mean ± SD. BMI: body mass index, MI: myocardial infarction, HF: heart failure, CVA: cerebrovascular accident, STEMI: ST-segment elevation myocardial infarction, CCB: calcium channel blocker, AECI: angiotensin-converting enzyme inhibitor, ARB: angiotensin receptor blocker, LVEF: left ventricular ejection fraction, CK-MB: creatine kinase myocardial band, Hs-CRP: high-sensitivity C-reactive protein, NT-pro-BNP: N-terminal pro-brain natriuretic peptide, ARU: aspirin reactivity unit, PRU: platelet reactivity unit.

During hospitalization, the ticagrelor or prasugrel group received an angiotensin-receptor blocker (ARB) more frequently than the clopidogrel group. Angiotensin-converting enzyme inhibitor (ACEI) was administered more frequently in the clopidogrel group than in the ticagrelor or prasugrel group. However, there were no significant differences in the drugs administered during hospitalization between the two groups after PSM (Table 1).

### Echocardiographic and laboratory findings

Hemoglobin level was higher in the ticagrelor or prasugrel group than in the clopidogrel group, and platelet reactivity unit was higher in the clopidogrel group than in the ticagrelor or prasugrel group. Echocardiography revealed no significant difference in LVEF between the two groups. After PSM, there was no difference in LVEF between the two groups (Table 1).

### Coronary angiographic findings

PCI was performed frequently via the transradial route in the ticagrelor or prasugrel group and via the transfemoral route in the clopidogrel group. According to the ACC/AHA classification of coronary lesions, B1/B2 type of lesions was common in the ticagrelor or prasugrel group, and C type of lesions was common in the clopidogrel group. As for the involved vessel, there were many single vessels in the ticagrelor or prasugrel group, and there were many multi- vessels in the clopidogrel group. There was no significant difference in TIMI flow between the two groups before and after the procedure. After PSM, there was no significant difference in coronary angiographic findings and procedural characteristics between the two groups (Table 2).

**Table 2:** Coronary angiographic findings and procedural characteristics of the clopidogrel and ticagrelor or prasugrel groups.

		Overall patients				After propensity score matching		
		(n = 4,696)				(n = 1,242)		
Variables		Clopidogrel group (n = 4,053)	Ticagrelor or prasugrel group (n = 643)		p-value	Clopidogrel group (n = 621)	Ticagrelor or prasugrel group (n = 621)	p-value
**Puncture route, n (%)**								
Transradial		1,409 (34.8)	298 (46.3)		<0.001	265 (42.7)	285 (45.9)	0.253
Transfemoral		2,644 (65.2)	345 (53.7)			356 (57.3)	336 (54.1)	
**ACC/AHA type, n (%)**								
A	78 (1.9)		4 (0.6)		0.001	13 (2.1)	4 (0.6)	0.055
B1/B2	1,917 (47.3)		364 (56.6)			321 (51.7)	357 (57.5)	
C	2,058 (50.8)		275 (42.8)			287 (46.2)	260 (41.9)	
**Target vessel, n (%)**								
LM	96 (2.4)		15 (2.3)		0.965	14 (2.3)	15 (2.4)	0.994
LAD	1,891 (46.7)		303 (47.1)			294 (47.3)	292 (47.0)	
RCA	697 (17.2)		114 (17.7)			114 (18.4)	112 (18.0)	
LCX	1,369 (33.8)		211 (32.8)			199 (32.0)	202 (32.5)	
**Number of involved vessel, n (%)**								
single vessel	1,847 (45.6)			356 (55.4)	<0.001	335 (53.9)	341 (54.9)	0.732
multi-vessel	2,206 (54.4)			287 (44.6)		286 (46.1)	280 (45.1)	
Pre-PCI TIMI flow ≤2, n (%)	2,906 (71.7)			484 (75.3)	0.060	448 (72.1)	470 (75.7)	0.155
Post-PCI TIMI flow 3, n (%)	3,938 (97.2)			627 (97.5)	0.618	607 (97.7)	605 (97.4)	0.712

Data are presented as number (%). ACC/AHA: American College of Cardiology/American Heart Association, LM: left main artery, LAD: left anterior descending artery, RCA: right coronary artery, LCX: left circumflex artery, TIMI: Thrombolysis in Myocardial Infarction.

### Hospitalization outcomes

Regarding complications during hospitalization, the clopidogrel group had a lower incidence of recurrent infarction and ischemia than the ticagrelor or prasugrel group. The ticagrelor or prasugrel group had a higher incidence of TIMI major bleeding than clopidogrel group. After PMS, there were no differences in the incidences of recurrent ischemia and infarction between the two groups. The ticagrelor or prasugrel group had higher TIMI major bleeding and TIMI minor or major bleeding than the clopidogrel group (Table 3).

**Table 3:** Complications during hospitalization of the clopidogrel and ticagrelor or prasugrel groups.

	Overall patients			After propensity score matching		
	(n = 4,696)			(n = 1,242)		
Variables	Clopidogrel group (n = 4,053)	Ticagrelor or prasugrel group (n = 643)	p-value	Clopidogrel group (n = 621)	Ticagrelor or prasugrel group (n = 621)	p-value
**In-hospital complications**						
Recurrent infarction	15 (0.4)	9 (1.4)	0.015	5 (0.8)	8 (1.3)	0.332
Recurrent ischemia	27 (0.7)	11 (1.7)	0.031	7 (1.1)	11 (1.7)	0.069
Stroke	60 (1.5)	11 (1.7)	0.657	6 (1.0)	10 (1.6)	0.314
TIMI minor bleeding	80 (2.0)	16 (2.5)	0.392	8 (1.3)	16 (2.6)	0.099
TIMI major bleeding	44 (1.1)	13 (2.0)	0.044	4 (0.6)	13 (2.1)	0.028
TIMI minor or major bleeding	112 (2.8)	26 (4.0)	0.074	12 (1.9)	26 (4.2)	0.021

Data are presented as number (%). TIMI: Thrombolysis in Myocardial Infarction.

### Clinical outcomes

After 3 years of follow-up, the incidences of cardiac death, recurrent MI, stroke, cerebral hemorrhage, CABG, and MACE were not significantly different between the two groups. Repeat PCI procedures were performed significantly more frequently in the clopidogrel group than in the ticagrelor or prasugrel group (10.5% vs. 7.3%, p = 0.012). After PSM, repeat PCI procedures were performed significantly more frequently in the clopidogrel group than in the ticagrelor or prasugrel group (12.2% vs. 7.6%, p = 0.006) (Table 4).

**Table 4:** Clinical outcomes in the clopidogrel and ticagrelor or prasugrel groups during a 3-year follow-up period.

	Overall patients			After propensity score matching		
	(n = 4,696)			(n = 1,242)		
Variables	Clopidogrel group (n = 4,053)	Ticagrelor or prasugrel group (n = 643)	p-value	Clopidogrel group (n = 621)	Ticagrelor or prasugrel group (n = 621)	p-value
MACE	885 (21.8)	135 (21.0)	0.631	134 (21.6)	127 (20.5)	0.626
NACE	903 (22.3)	140 (21.8)	0.774	137 (22.1)	132 (21.3)	0.731
Cardiac death	325 (8.0)	65 (10.1)	0.074	44 (7.1)	60 (9.7)	0.101
Recurrent MI	147 (3.6)	22 (3.4)	0.795	21 (3.4)	21 (3.4)	1.000
Repeat PCI	426 (10.5)	47 (7.3)	0.012	76 (12.2)	47 (7.6)	0.006
Stroke	49 (1.2)	6 (0.9)	0.546	9 (1.4)	6 (1.0)	0.436
TIMI major bleeding	18 (0.4)	5 (0.8)	0.260	3 (0.5)	5 (0.8)	0.478
CABG	18 (0.4)	3 (0.5)	0.937	2 (0.3)	3 (0.5)	0.654

Data are presented as number (%). NACE: net adverse cardiac event, MACE: major adverse cardiac events, MI: myocardial infarction, PCI: percutaneous coronary intervention, TIMI: Thrombolysis in Myocardial Infarction, CABG: coronary artery bypass graft.

**Fig 2:**
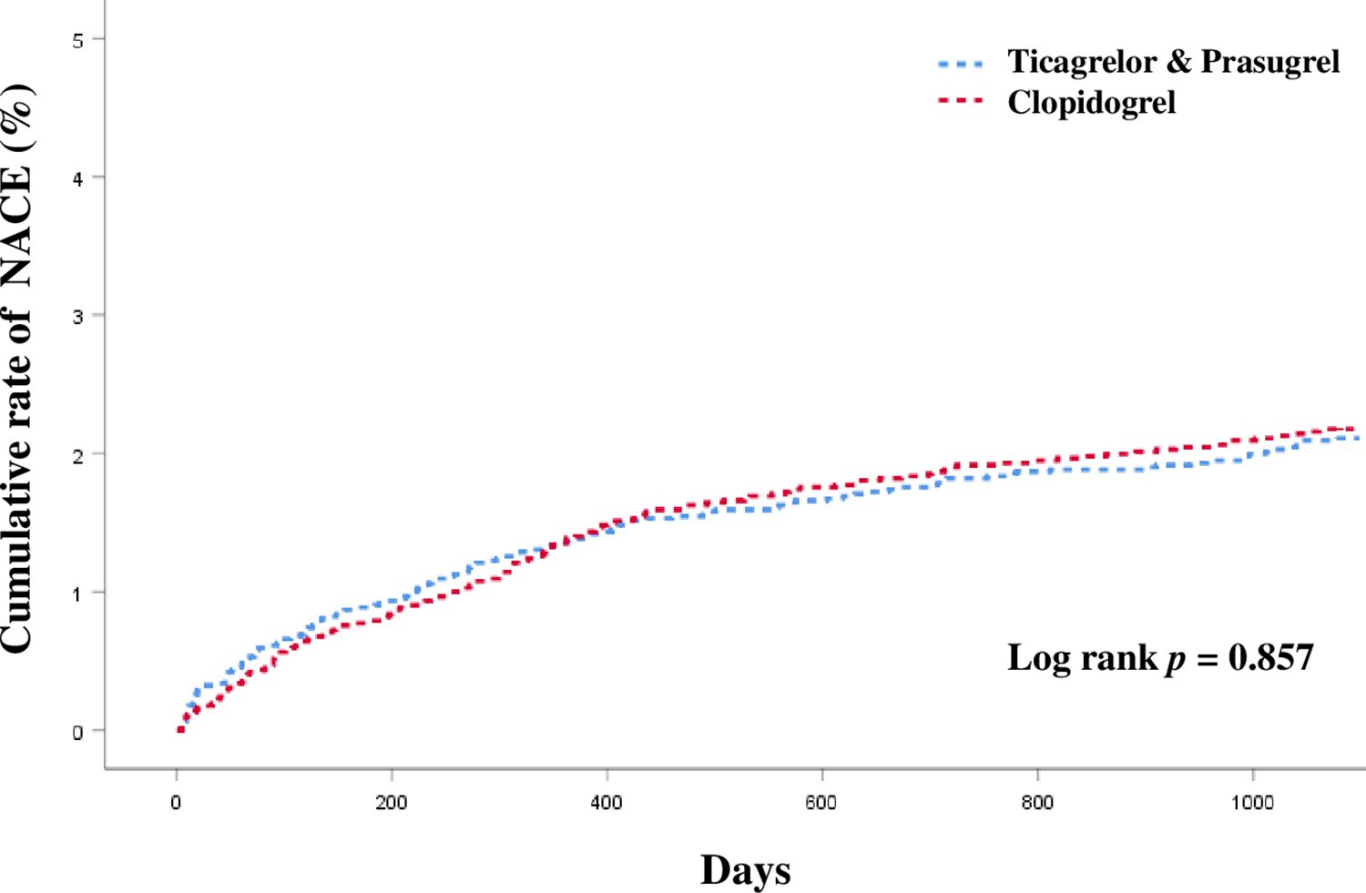
Kaplan–Meier curve for net adverse cardiac event (NACE) in clopidogerl group and ticagrelor or prasugrel group.

Kaplan-Meier survival analysis revealed no significant differences in NACE, including major cardiac events and major bleeding, between the two groups (Fig 2).

### Predictive factors of 3-year NACE

In patients with myocardial infarction who took ticagrelor, prasugrel, and clopidogrel, the predictors of NACE were an LVEF of < 40%, Hb level of < 12 mg/dL, multi-vessel disease, and creatinine levels ≥ 1.3 mg/dL (Table 5).

**Table 5:** Cox regression analysis for independent predictors of 3-year NACE in patients with acute myocardial infarction.

	Patients with MI (n = 1,242)					
	Univariate analysis			Multivariate analysis		
	HR	95% CI	p-value	HR	95% CI	p-value
Female sex	1.476	1.130–1.928	0.004			
clopidogrel	1.022	0.804–1.299	0.857			
Age ≥ 65 years	1.709	1.345–2.172	<0.001			
Killip class 3	1.814	1.301–2.531	<0.001			
Hypertension	1.537	1.202–1.964	0.001			
Diabetes	1.246	0.978–1.587	0.075			
Femoral puncture	1.281	1.001–1.639	0.049			
LVEF < 40 %	2.116	1.569–2.853	<0.001	1.430	1.040–1.966	0.028
Creatinine > 1.3 mg/dL	3.082	2.356–4.032	<0.001	2.098	1.529–2.879	<0.001
Hemoglobin level < 12 mg/dL	2.427	1.874–3.143	<0.001	1.395	1.015–1.919	0.040
Multi-vessel disease	1.728	1.356–2.202	<0.001	1.487	1.162–1.903	0.002

Hazard ratios were calculated using Cox regression analysis. NACE: net adverse cardiac event, HR: hazard ratio, CI: confidence interval, LVEF: left ventricular ejection fraction.

### Exploratory outcomes

**Fig 3:**
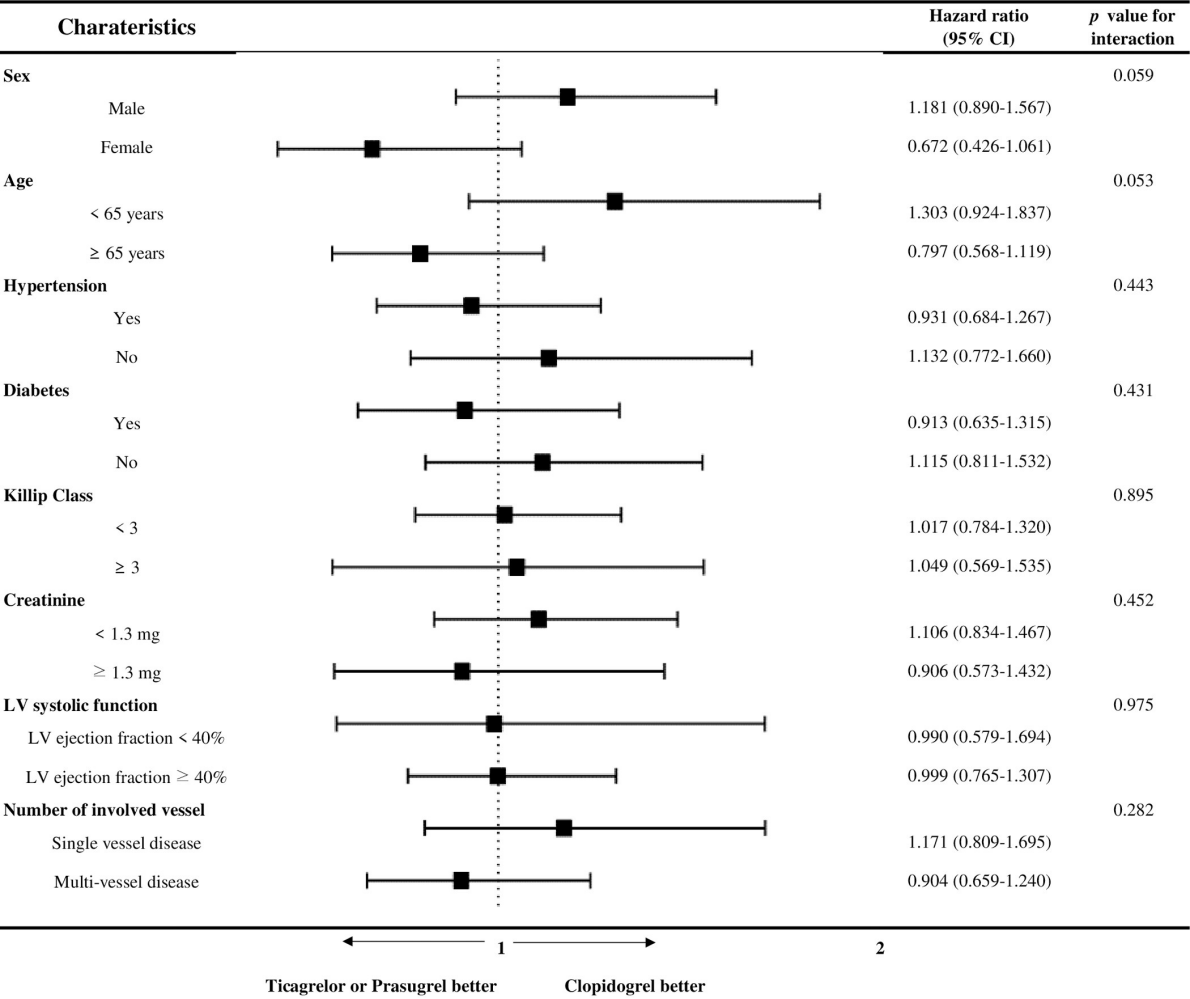
Forest plot of exploratory subgroup analysis between clopidogrel group and ticagrelor or prasugrel group for NACE (net adverse cardiac event). CI: confidence interval, LV: left ventricular.

Exploratory subgroup analyses of the clopidogrel group and ticagrelor or prasugrel group accounted for the HR for the incidence of NACE between patients receiving clopidogrel and those receiving ticagrelor. The treatment effect of ticagrelor or prasugrel compared to clopidogrel was consistent across all exploratory subgroups without statistically significant treatment-by-subgroup interactions (Fig 3).

## Discussion

In this study, the clinical outcomes of patients with myocardial infarction who underwent PCI and were taking antiplatelet drugs for 24 months without changing or discontinuing the drug were assessed. This study also investigated the prognosis in patients with AMI taking clopidogrel and a P2Y_12_ inhibitor, ticagrelor or prasugrel, for more than 24 months. Subjects were observed for a total of 36 months, including a 12-month follow-up period. The clopidogrel group had a higher incidence of recurrent PCI than the ticagrelor or prasugrel group. Incidence of major bleeding was not different between the two groups.

In the TRITON-TIMI 38 study, the effect of prasugrel and clopidogrel administration was compared in patients with STEMI who underwent PCI. The prognosis of patients taking prasugrel was good for cardiovascular death, myocardial infarction, stroke, and stent thrombosis during 15 months of follow-up [20]. In the PLATO study comparing the efficacy of ticagrelor and clopidogrel in patients with ACS, the incidence of CVD, MI, stroke, RI (recurrent ischemia), SRI (severe recurrent ischemia), and TIA (transient ischemic attack) was lower in patients taking ticagrelor than in those taking clopidogrel. There was no difference in major bleeding between the two groups [21]. The current ACCF/AHA and ESC guidelines for antiplatelet therapy recommend ticagrelor or prasugrel as first-line treatment in patients with ACS after PCI [6, 7]. However, the patients in previous randomized clinical trials comparing the effects of the next-generation P2Y_12_ inhibitor and clopidogrel were mostly Westerners and studies targeting East Asians are lacking. Similar to the results of this study, a 12-month follow-up study of patients with ACS taking ticagrelor or clopidogrel in Korea reported that the incidence of clinically significant bleeding was significantly higher in the ticagrelor group than in the clopidogrel group. The incidence of death from cardiovascular causes, myocardial infarction, or stroke was not significantly different between the ticagrelor and clopidogrel groups [22].

A study comparing the effect of clopidogrel and prasugrel in patients with myocardial infarction in Korea showed that there was no significant difference in cardiac death, MI, stroke, or target vessel revascularization between the two groups. Bleeding was significantly higher in the prasugrel group than in the clopidogrel group [23]. In a study conducted on Japanese, Korean, and Taiwanese patients with ACS comparing the effect of ticagrelor and clopidogrel reported that there was a significant difference in the safety or efficacy of clopidogrel and ticagrelor [24].

Recent studies have shown that Westerners and East Asians have different effect-risk ratios for P2Y_12_ inhibitors [25, 26]. Compared with Westerners, East Asians usually have a lower risk of ischemic events and a higher risk of gastrointestinal bleeding or hemorrhagic stroke during antithrombotic treatment, which is called the East Asian paradox [27]. These results suggest that the efficacy of platelet suppression treatment may differ due to racial factors.

In addition, the ACC/AHA and ESC guidelines recommend maintaining DAPT for at least 12 months [6, 7]. A study on the duration of DAPT compared three groups taking aspirin and prasugrel or ticagrelor for < 12 months, 12 months, and > 12 months. MACE decreased significantly with increasing DAPT duration [28]. The recommendations for the use of P2Y_12_ receptor antagonists by the 2020 Asian Pacific Society of Cardiology explain that a phased reduction in the duration of DAPT administration, discontinuation, or continuation of treatment over 12 months in patients after PCI should be individualized considering the risk of ischemia and bleeding in each patient [29]. Contrary to the general guideline [6, 7] recommendations for DAPT in the first 12 months after ACS, long-term DAPT requires a risk-benefit assessment in individual patients. One verified algorithm to assess long-term risk is DAPT score. A new risk score (the “DAPT score”) suggested in the ACC/AHA guidelines evaluates the risk-benefit ratio of extended DAPT considering age, smoking habits, diabetes, MI at presentation, PCI or prior MI, small stent diameter (< 3 mm), paclitaxel-eluting stents, chronic heart failure or LVEF < 30%, and vein graft stent [30]. The PRAISE risk score stratifies patient risk using machine-based learning and provides a suitable DAPT for patients based on the bleeding and ischemic profiles of ACS patients [31]. In 2020, the KAMIR-DAPT score was introduced in the domestic expert consensus document on AMI pharmacotherapy. The use of low-dose prasugrel or ticagrelor may be considered based on risk-benefit in Korean patients with myocardial infarction. Additionally, the P2Y_12_ inhibitor can be determined using the KAMIR-DAPT score that is tailored to the characteristics of Korean patients with AMI at risk of ischemia and bleeding events [11].

There are antiplatelet treatment guidelines for patients with myocardial infarction [6], and each country and each ethnic group has recommended antiplatelet drugs [11, 29]. Individualized antiplatelet therapy should be administered to patients considering the complexity of individual pathophysiology and reactivity to antiplatelet agents. In addition, further studies on the dosage and duration of antiplatelet drugs are needed. Additional research is necessary to select the component, dose, and duration of an antiplatelet agent individualized to each clinical myocardial infarction patient.

This study has some limitations. It was based on data collected from a cohort study, There is a lack of characteristics of DAPT strategies, such as the presence of chronic kidney disease, branching therapy, chronic overall occlusion rate, and severely calcified lesions. PSM analysis was performed based on registry data, but other variables affecting clinical practice in AMI were not included. Although most of the patients took the standard drug dose, these could have been adjusted during the follow-up period in some patients. Therefore, further analysis is required according to dose. Our registry evaluated bleeding according to the criteria defined by the TIMI and not according to the BARC bleeding criteria. Finally, TIMI major and minor bleeding were included as complications during hospitalization, but only major bleeding was assessed during the follow-up period. Actual bleeding might have been underestimated because not all events were included in the registry database.

In conclusion, DAPT should be individualized and customized because the risk of ischemia and bleeding events differs across patients with myocardial infarction. In addition, the type and dose of P2Y_12_ inhibitor and treatment duration should be determined for each individual with myocardial infarction.
